# Dairy cow performance is associated with longitudinal microRNA profiles

**DOI:** 10.1371/journal.pone.0328765

**Published:** 2025-08-01

**Authors:** Madison MacLeay, Francesc Xavier Donadeu, Georgios Banos

**Affiliations:** 1 The Roslin Institute and Royal (Dick) School of Veterinary Studies, University of Edinburgh, Easter Bush, Midlothian, United Kingdom; 2 Department of Animal and Veterinary Sciences, Scotland’s Rural College, Roslin Institute Building, Easter Bush, Midlothian, United Kingdom; National Bureau of Animal Genetic Resources, INDIA

## Abstract

Modern high producing dairy cows are still affected by poor fertility and disease, despite improvements achieved through genetic selection programs. Additional biomarkers of health and performance traits in cattle could enhance animal welfare and profitability by allowing farmers to cull animals before problems occur. We performed pilot investigations of plasma microRNA (miRNA) profiles during early life as potential biomarkers associated with future performance in dairy cows. The latter included survival to two years of age, age at first calving, yield of milk, fat and protein, mastitis and lameness traits, conception rate, number of services per conception, and calving interval. Using qPCR, we obtained longitudinal measurements and ratios involving nine miRNAs (miR-126-3p, miR-127, miR-142-5p, miR-154b, miR-27b, miR-30c-5p, miR-34a, miR-363, miR-425-3p) in plasma samples from three age groups: calves (<1 month), heifers (14–23 months), and first lactation cows (29–35 months). Changes in miR-126-3p from calf to first lactation cow were associated with first lactation milk yield and second lactation milk somatic cell count (an udder health indicator). Moreover, the miR-127 to miR-30c-5p ratio in cows was associated with milk fat and protein yield in the first two lactations, whereas miR-142-5p levels and several miRNA ratios involving this miRNA, were associated with second calving interval (a cow fertility trait). Our results identified novel early life biomarkers that warrant further investigation to determine whether they may predict dairy cattle performance.

## Introduction

High producing dairy cows present higher incidence of both poor fertility and disease. There is an antagonistic relationship of milk yield with animal health and fertility, partly due to negative energy balance in early lactation, when nutrients and reserves are allocated to elevated milk production. Animals with greater negative energy balance and loss of body condition take longer to return to oestrus after calving and have lower conception rates [1,2]. Adding relevant fertility and health traits to genetic selection programs has helped, but progress is slow and there is room for further improvement [3].

Gates (2013) reported that less than 7% of dairy heifers calve by the goal age of 24 months, and calving interval averaged 428 days instead of the one-year goal [4]. This has knock-on effects on later cow performance and farm profitability. First calving at around two years of age is associated with better subsequent fertility and milk production compared to later or, in fact, earlier ages [5,6]. Moreover, a significant proportion of dairy cows are culled, primarily due to disease and poor fertility [7]. For example, in the UK, 20% of dairy heifers are culled before the first lactation [8], whereas in the USA, an average annual cull rate of cows of 38% has been reported [9].

Disease has a major impact on cow welfare, survival, fertility, and production. Mastitis leads to reduced milk production, lower conception and pregnancy rates, higher pregnancy losses, and involuntary culling, in addition to further profit loss because milk is discarded after treatment [10]. Lameness also diminishes milk production, affects nutrition by reducing time spent feeding, and reduces fertility [11]. Other postpartum diseases including endometritis and disorders relating to nutrition, such as ketosis and hypocalcaemia, may delay post-partum return to oestrus and subsequent conception [12,13]. Reducing disease risk in dairy cows would improve cow health and welfare. Accurate prediction of future performance would also improve farm profitability [9], and existing culling decision support tools would benefit from more detailed input [14].

There is a wealth of promising research on the utility of microRNAs (miRNAs) as biomarkers of animal disease, fertility, milk production, and survival. Circulating miRNAs are ideal biomarkers; they can be exported from cells and show enhanced stability in circulation due to association with lipids and exosomes [15]. Mastitis and other infections in cattle are reportedly associated with distinct miRNA profiles in body fluids [16,17]. Such profiles also vary during the oestrus cycle [18], pregnancy [19,20] and lactation [21,22] in cows. miRNAs have also been associated with puberty onset in chicken and rats [23,24], though in cattle there are very few similar reports [25]. Studies of milk content revealed differentially expressed miRNAs in animals with varying milk fat [26] and casein content [27]. miRNAs are integral in growth and developmental processes, and circulating miRNA levels vary with age [28,29]. Use of miRNAs as risk biomarkers for later outcomes including cardiovascular disease, metabolic syndrome, and even lifespan has been proposed in humans [30–32].

In the present study, we hypothesized that variation in miRNA profiles during early life may be associated with future performance and survival in dairy cattle. Our previous work used longitudinal measurements of plasma miRNA to investigate how miRNAs change with age and whether they are associated with early life performance, such as calf health and growth [33]. Here, we further investigated the shared functions and correlations among the previously studied miRNAs and assess associations between miRNA expression profiles (single miRNAs, miRNA combinations, and longitudinal expression changes) in young animals and later cow performance traits, including milk production, fertility, health, and survival.

## Materials and methods

### Ethics approval

All animal procedures were performed with approval from The Roslin Institute (University of Edinburgh) Animal Welfare and Ethical Review Board and following the UK Animals (Scientific Procedures) Act, 1986. This study was conducted and reported in accordance with ARRIVE guidelines. Consent to access animal facilities was granted by the University of Edinburgh.

### Experimental animals and sample collection

Blood plasma samples were available from cows born at Langhill Farm (University of Edinburgh, Scotland) during the winter of 2017/18 (n = 119) and 2018/19 (n = 108). By design, cows at this farm calve over the winter months each year, thus the winter of calving was used as a cohort factor.

Samples were collected at three timepoints on each animal corresponding to three distinct age groups: calves ≤1 month of age (n = 227), heifers aged 14–23 months (n = 209), and first lactation cows aged 29–35 months (n = 98). Samples were transported on ice from the farm to the lab, and plasma was separated the same day. To isolate the plasma, whole blood was centrifuged at 1900xg at 4°C for 10 minutes. The supernatant was centrifuged again at 1900xg at 4°C for 10 minutes. The supernatant was removed and stored at −80°C until use. Haemolysis in blood plasma samples causes release of intracellular miRNAs, thus can alter levels of several miRNAs and influence quantification accuracy. To account for this, samples were tested for haemolysis based on absorbance at 414nm as measured on a Nanodrop 1000 spectrophotometer, which was shown to be one of the more sensitive methods, after miRNA ratio [34]. In the present study, samples with readings over 0.2 were considered significantly haemolysed and removed from further study, as recommended by Kirschner and colleagues [35]. All identified readings above this threshold were also outliers (>2 standard deviations above the mean).

### Quantification of circulating miRNAs

The miRNAs of interest had been identified in a previous study using PCR array profiling of animals that varied in different early life performance traits [33]. Briefly, Generalized Linear Mixed Models and t-tests were used to identify miRNAs in the PCR arrays that were significantly associated with early life growth, fertility, and health. Nine candidate miRNAs were chosen for further analysis as these displayed statistically significant associations with multiple early life performance traits, and were also considered in the present study: miR-126-3p, miR-127, miR-142-5p, miR-154b, miR-27b, miR-30c, miR-34a, miR-363, and miR-425-3p. These were quantified in plasma from 91 animals at all age groups (calf, heifer, cow). RNA extraction and RT-qPCR was performed [33,36] using miRCURY LNA system (Qiagen) and a Stratagene Mx3000P qPCR machine (Agilent, USA). The mean expression of two miRNAs, bta-miR-20a and bta-miR-106a, was used to normalize all qPCR data, as in our previous study [33].

### Relationships among miRNAs

We explored interdependencies between the nine studied miRNAs to inform the number of independent groups to account for when adjusting p-values for multiple testing in subsequent analyses. As functions for these miRNAs have not been experimentally validated in cattle, they cannot reliably inform the number of independent groups to consider. Therefore, we explored the relationships among these miRNAs in several ways to determine the most appropriate number of independent tests, as well as provide additional insight into their expression as a group: pairwise correlations among the miRNAs and clustering analyses. Spearman pairwise correlations between miRNA levels across all age groups were calculated using R v4.2.2. A multiple factor analysis (MFA) was performed followed by K means clustering to group miRNAs based on their levels in each of the three age groups. To this effect, normalized miRNA levels were adjusted for batch differences (different RNA extraction and RT-qPCR runs) by adding the residuals from a model of batch on each miRNA to the overall mean of that miRNA. Subsequently, miRNA measurements were binary log-transformed to ensure a normal distribution [37]. There were missing values because some sampled animals had been culled or died before all samples could be collected or their samples were of poor quality, mainly due to haemolysis, PCR failure or >1.5Ct difference between replicates. Therefore, multiple imputation was performed using the missMDA package in R and the function, imputeMFA(). The FactoMineR package was used for MFA and the resulting coordinates were used for K means clustering. Four clusters were chosen by visual inspection. To determine whether the miRNAs cluster based on the putative gene pathways they target, miRPath v3.0 was used. This software was used previously to identify the gene pathways targeted by the human orthologues of the miRNAs based on Tarbase v7.0 [33]. The software performs hierarchical clustering of the miRNAs and produces a dendrogram (‘Targeted pathways clusters’ option) [38,39]. Bta-miR-154b is ruminant specific so was not included in this analysis.

Ratios between all possible pairs of miRNAs, irrespective of order, were also calculated for further investigation.

### Hierarchical clustering of animals based on miRNA levels

Principal Component Analysis and agglomerative hierarchical clustering of animals with average linkage were performed on batch-adjusted log-transformed miRNA levels, as measured across all age groups. These analyses were repeated separately for each of the nine studied miRNAs using the HCPC() function from FactoMineR in R. The number of clusters was determined automatically.

### Associations between miRNA profiles and cow performance traits

Each cow had records on multiple performance traits, which included milk production (first and second lactation yield of milk, fat, and protein), fertility (conception rate [CR], number of services per conception [S/C], age at first calving [AFC], calving interval), health (incidence of mastitis, incidence of lameness, median lactation milk somatic cell count [SCC]), and survival to two years of age.

The miRNA profiles considered for association with cow performance included measurements of single miRNA levels in each of the three age groups (calf, heifer, cow); ratios between all miRNA pairs within age group; the cluster the animal belonged to for a single miRNA, as identified using hierarchical clustering; and fold change in levels of each miRNA between two age groups.

Univariate general(ised) linear mixed models were applied to each cow trait, separately, including each miRNA profile measure as a fixed term. The other terms in the models were determined by backwards selection to include factors with a significant effect (P < 0.05) on each cow trait. All models included the cohort of calving (or birth, for AFC and survival to two years) effect. Additional terms included length of lactation (days) for milk production traits; the calendar month of calving for S/C, CR, and calving interval; calendar month of birth for AFC; the age of calving (days) for CR and calving interval; and the number of episodes of infection in the first 12 months of life for survival to two years. The animal effect including pedigree was fitted as a random effect in all analyses. The miRNA measurement effect estimated with these analyses would then be reflective of the association of the respective miRNA, ratio, cluster, or fold change with performance adjusted for all other effects in the model.

Prior to the statistical analysis, SCC records were log-transformed to ensure normal distribution. Linear models were then applied to all milk production traits, SCC, calving interval, and AFC. A Poisson function was applied to S/C. Binomial functions were applied to CR, incidence of mastitis, incidence of lameness, and survival to two years of age.

The nominal statistical significance level (P = 0.05) was corrected for multiple testing using the Bonferroni method, considering 16 independent tests. This number was based on the relationships among miRNAs previously described, where the K means clustering and the miRPath dendrogram revealed four distinct miRNA groups; and four groups of cow trait functions related to milk production, fertility, health, and survival, respectively.

## Results

### Relationships among miRNAs

Pairwise correlations between all nine miRNAs revealed a total of 23 significant correlations, with respective coefficients ranging from 0.26 to 0.88 (Table 1). The miRNA with the fewest statistically significant (P < 0.05) correlations was miR-27b, which was positively correlated with miR-34a (r = 0.42) and miR-126-3p (r = 0.42). The miRNA with the most significant correlations was miR-126-3p, which was correlated with all other candidate miRNAs. This correlation was positive with miR-27b, miR-30c-5p, miR-34a, miR-142-5p, and miR-425-3p, and negative with miR-127, miR-154b, and miR-363.

**Table 1:** Spearman pairwise correlation coefficients between the 9 candidate miRNAs. An asterisk (*) indicates P < 0.05.

	miR-142-5p	miR-425-3p	miR-126-3p	miR-154b	miR-27b	miR-34a	miR-363	miR-127
miR-425-3p	0.82*							
miR-126-3p	0.41*	0.37*						
miR-154b	−0.34*	−0.11	−0.58*					
miR-27b	0.00	0.05	0.42*	−0.14				
miR-34a	0.22	0.18	0.57*	−0.30*	0.42*			
miR-363	−0.23	−0.28*	−0.57*	0.57*	0.08	−0.20		
miR-127	−0.13	0.07	−0.48*	0.88*	−0.16	−0.31*	0.50*	
miR-30c-5p	0.53*	0.42*	0.58*	−0.34*	0.18	0.50*	−0.26*	−0.26*

**Fig 1:**
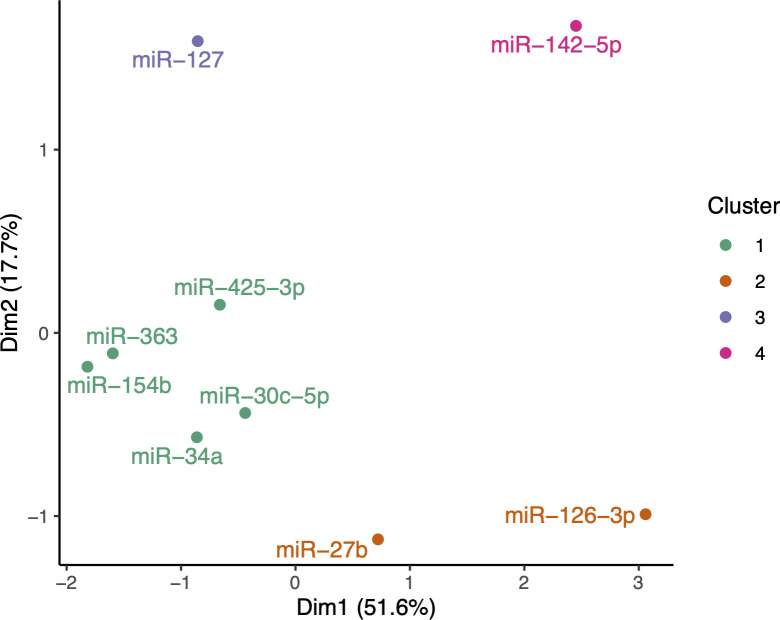
Biplot from the multiple factor analysis (MFA). This shows the first two dimensions (with proportion of variance explained) and each miRNA coloured by K means cluster.

Results from the multiple factor analysis (MFA; Fig 1) suggested that heifer and cow miRNA levels were more similar to each other than to the calf miRNA levels, since the former had higher contributions to the first principal component (36.5% and 36.8%, respectively) and the calf levels had the highest contribution to the second principal component (59.7%). This reflects the greater changes in miRNA expression levels between calves and heifers than between heifers and cows, as previously reported [28,33]. miRNAs grouped in four clusters, one containing five miRNAs and another containing two (miR-27b and miR-126-3p), whilst miR-127 and miR-142-5p each formed their own cluster.

**Fig 2:**
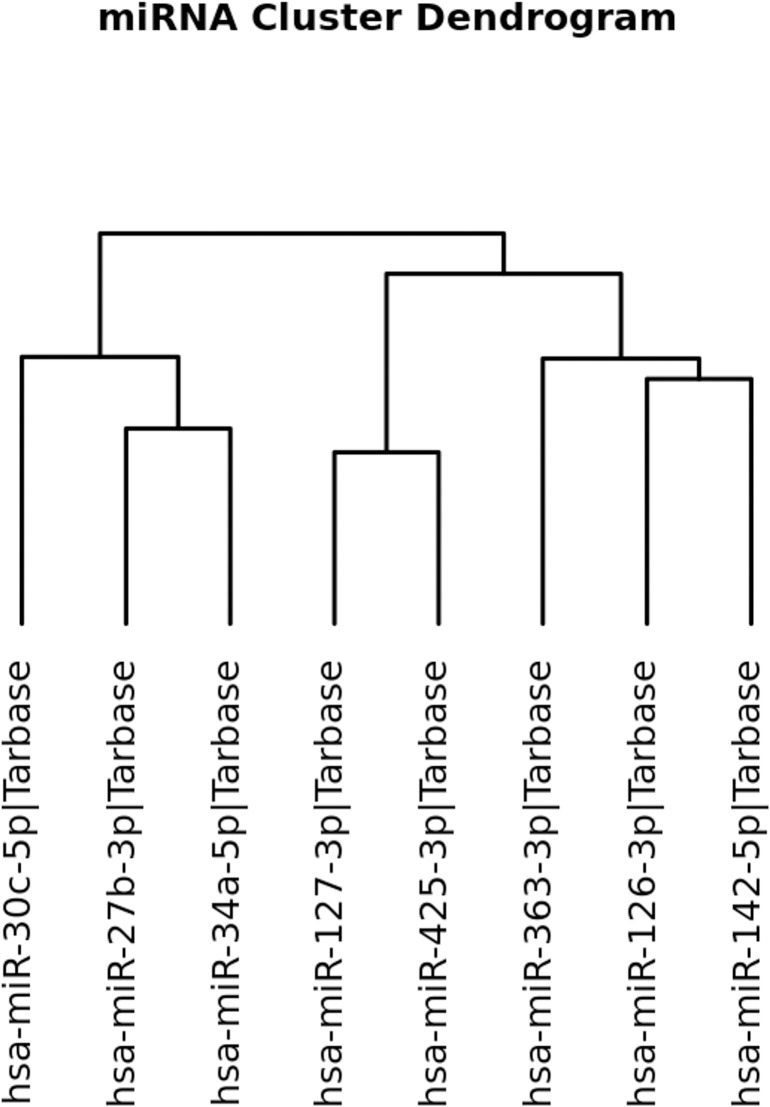
Dendrogram for eight miRNAs based on gene pathways targeted in humans.

The dendrogram generated by miRPath analysis of the studied miRNAs is shown in Fig 2. The gene pathways targeted by these miRNAs was also previously reported [33]. Only eight of the bovine miRNAs had human counterparts, and among these, there were three clusters. Since miR-154b is ruminant-specific and could not be included in these three clusters, there were four groups of miRNAs to consider as independent during the ensuing downstream analyses.

### Hierarchical clustering of animals based on miRNA levels

**Fig 3:**
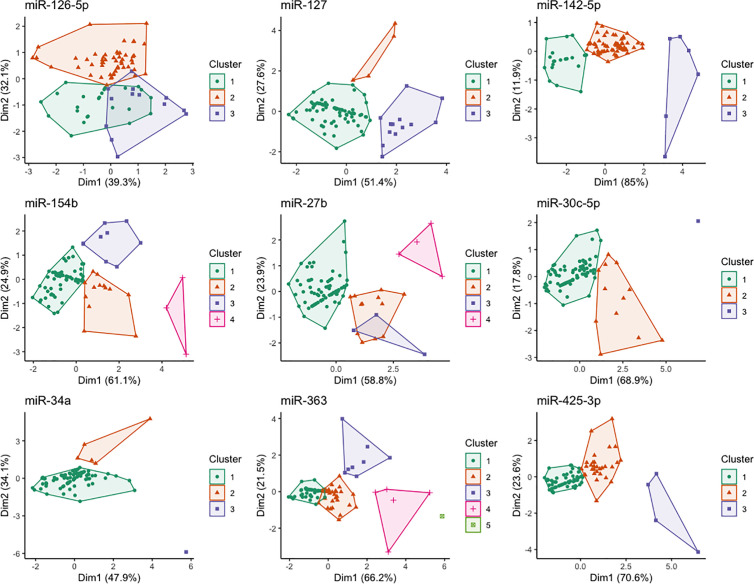
Principal component analysis biplots of the first two dimensions (with percentage of variance explained) for each miRNA, with individual animals shaded by cluster.

**Fig 4:**
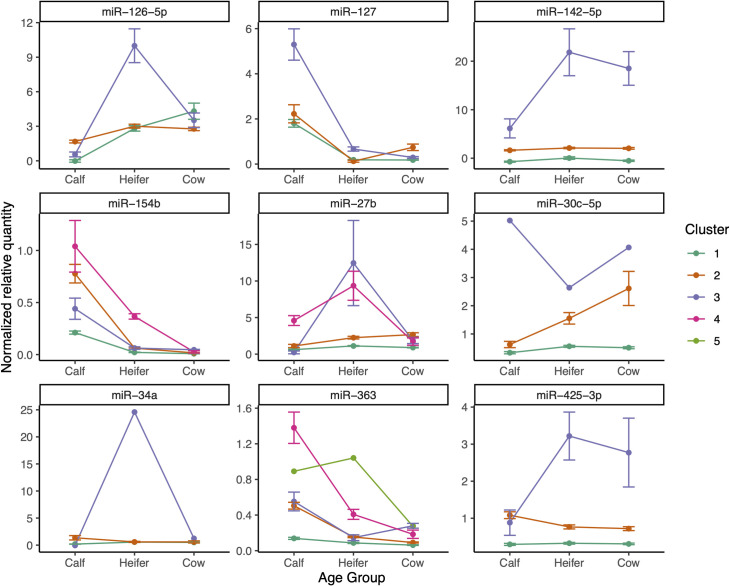
Mean (SE) miRNA levels in the three age groups for each cluster, for each miRNA.

Fig 3 shows the biplots from the Principal Component Analysis of animals, shaded by cluster, for each miRNA. The size of clusters varied; for many miRNAs (e.g., miR-30c-5p, miR-34a, miR-363) there was an outlier that formed its own cluster. Clusters of only 3–4 animals were common. Fig 4 shows how the miRNA levels change with age for each cluster. Usually, the miRNA levels in different clusters changed in the same direction, but the magnitude of change varied. However, for some miRNAs, the direction of change with age was different between clusters. For example, for miR-126-3p and miR-27b, some clusters showed a steady increase in levels over time while others showed a peak at the heifer stage. Similarly, for miR-127 and miR-363, most clusters showed a decrease in miRNA levels over time, but there was one cluster that showed a subsequent increase at the cow stage. For miR-425-3p, some clusters showed a decrease from calf to heifer while others showed an increase.

### Associations between miRNA profiles and cow performance traits

The miRNA profile measurements considered here were 1) expression levels in calves, heifers, and cows, 2) pairwise miRNA ratios in each of the age groups, 3) fold change from one age group to another, and 4) a categorical variable for the cluster an animal belongs to for each miRNA, as described in the previous section. The effect of these miRNA variables on multiple cow performance traits was assessed.

The associations that remained statistically significant (P < 0.05) or had borderline statistical trends (0.05 < P < 0.1) after adjusting for multiple testing are listed in Table 2. Corresponding plots for the significant associations (P < 0.05) can be seen in S1 File. Most significant associations involved miRNA ratios and pertained to milk production traits and calving interval. Among individual miRNAs, there was a significant positive association between miR-142-5p measured at the cow age group and second calving interval (from second to third calving of the cow). Levels of miR-126-3p in calves was associated with elevated SCC in first lactation, implying higher (sub)clinical mastitis incidence, whilst miR-30c-5p in calves was associated with decreased milk yield in second lactation. Calf to cow fold change in miR-126-3p was significantly associated with lower milk yield across the first two lactations. Animal cluster based on this miRNA was significantly associated with log median SCC in the second lactation. Cluster 1 had cows with the lowest median SCC, alongside the lowest calf miR-126-3p levels and the greatest cow miR-126-3p levels. Cluster 3 had the highest median SCC, with the highest heifer miR-126-3p levels and intermediate calf and cow levels. No significant associations were identified for S/C, CR, mastitis, lameness, survival to two years of age, or AFC.

**Table 2:** Associations between miRNA measurements and cow performance traits that remained statistically significant (P < 0.05, indicated by an asterisk *) or showed statistical trend (0.05 < P < 0.10) after adjusting for multiple testing, with effect size (SE). Associated Fig numbers are as labelled in S1 File.

miRNA/Ratio	miRNA measurement	Trait of interest	Effect (SE)	S1 File Fig
**Lactation 1**				
miR-127: miR-30c-5p	cow	Protein yield (kg)	−22.8 (6.4) *	S1
miR-127: miR-30c-5p	cow	Fat yield (kg)	−26.8 (8.4) *	S2
miR-127: miR-30c-5p	cow	Milk yield (kg)	−598.1 (203.6)	
miR-126-3p	calf	Log median SCC	0.27 (0.09)	
miR-126-3p	calf - > cow fold change	Milk yield (kg)	−651.8 (214)	
**Lactation 2**				
miR-126-3p: miR-27b	cow	Calving interval (days)	20.5 (3.8) *	S3
miR-142-5p	cow	Calving interval (days)	21.8 (5.5) *	S4
miR-142-5p: miR-27b	cow	Calving interval (days)	15.6 (4.5) *	S5
miR-142-5p: miR-363	cow	Calving interval (days)	18.1 (5) *	S6
miR-425-3p: miR-27b	cow	Calving interval (days)	23.5 (3.9) *	S7
miR-154b: miR-127	cow	Protein yield (kg)	31.8 (9) *	S8
miR-363: miR-127	cow	Log median SCC	−0.70 (0.20) *	S9
miR-126-3p	cluster	Log median SCC	2: 0.40 (0.23) * 3: 1.19 (0.29)	S10
miR-30c-5p	calf	Milk yield (kg)	−507.9 (172.1)	
miR-154b: miR-363	cow	Log median SCC	0.32 (0.11)	
**Combined lactations 1 and 2**				
miR-126-3p	calf - > cow fold change	Milk yield (kg)	−571 (189) *	S11
miR-127: miR-30c-5p	cow	Fat yield (kg)	−22.4 (6.7) *	S12
miR-127: miR-30c-5p	cow	Protein yield (kg)	−17.5 (5.6) *	S13
miR-127: miR-30c-5p	cow	Milk yield (kg)	−487.1 (171.3)	

## Discussion

This study aimed to identify novel associations between plasma profiles of nine miRNAs and cow performance traits related to milk production, fertility, health, and survival. We also investigated how these miRNAs may interact with each other, using correlation estimates, multiple factor analysis, and putative gene pathways targeted. The results deepened our understanding of how expression of these miRNAs changes throughout early life in dairy cattle and demonstrated for the first time that miRNA ratios and longitudinal miRNA profiles are associated with cow performance.

Multiple miRNA ratios were associated with cow traits of interest, suggesting a potential synergistic role in performance traits. miRNA ratios have not been widely studied outside cancer research in humans [40]. In the present study, most associations were between the second calving interval, a cow fertility trait manifested during her second lactation, and miRNA ratios measured prior to this, so these miRNAs have potential to predict later life performance. For example, two ratios including miR-142-5p (miR-142-5p: miR-27b and miR-142-5p: miR-363) were unfavourably associated with calving interval, implying delays in cow conception after her second calving. Individual levels of miR-142-5p were also adversely associated with the same trait. This miRNA is expressed in white blood cells [41,42] as well as lymph nodes and spleen [33]. Multiple studies have suggested miR-142-5p as a candidate early mastitis biomarker [43–45] in cattle. Lipopolysaccharide stimulation of bovine endometrial stromal cells caused upregulation of miR-142-5p, suggesting involvement with endometritis [46]. Mastitis, endometritis, and other postpartum infections may influence conception rate and, consequently, calving interval [10,12]. High levels of miR-142-5p may indicate infection that lengthens post-partum return to ovulation and oestrus and lowers chances of conception. This miRNA has also been associated with milk fat metabolism in goats [47] and cattle [48]. Therefore, altered metabolism may be at play, perhaps influenced by the presence of disease, and may affect calving interval [49].

Ratios involving miR-363 were associated with health and fertility traits. Similarly to miR-142-5p, miR-363 is found in the bovine spleen and lymph node [33], and has been linked with inflammation [50]. This miRNA is reportedly downregulated in bovine serum during Johne’s disease episodes [51], and has been linked with reduced endothelial cell inflammation [52,53]. This supports certain associations with udder inflammation found in the present study, namely, those of cow ratios miR-363: miR-127 and miR-154b: miR-363 with lower and higher second lactation median SCC, respectively. Milk SCC levels are a widely accepted indicator of udder infection including both clinical and subclinical mastitis. However, the present study did not identify any significant miRNA associations with clinical mastitis, probably due to very few clinical episodes that are routinely recorded in the herd. Studying a larger sample and additional clinical disease data would be of interest in this regard.

There was a significant association between second calving interval and the ratios miR-126-3p: miR-27b and miR-425-3p: miR-27b. miR-425-3p has been linked to endometritis in one previous study in cows [54], which is a condition that may lead to reduced conception rates and longer calving intervals. miR-27b has multiple roles with the potential to influence fertility and is broadly conserved among mammals. This is one of the most highly expressed miRNAs in many bovine tissues [19,44,55,56] and was differentially expressed in one previous study on mastitis in cattle [57]. Additional studies have linked this miRNA to adipogenesis in humans [58], mice [59], and sheep [60]. miR-27b is also reportedly involved in milk fat synthesis in goat mammary cells [61]. This miRNA may therefore be involved in metabolic disorders, which can impact calving interval as discussed above.

The ratio miR-127: miR-30c-5p was negatively associated with milk production traits in the first lactation and combined first and second lactations. Moreover, we saw a statistical trend between calf levels of miR-30c-5p and second lactation milk yield. In humans, miR-30c-5p has been primarily linked to lipid metabolism and adipogenesis [62,63]. There are fewer studies in livestock, but this miRNA is reportedly differentially expressed in beef cattle with high and low intramuscular fat [64], and is upregulated during adipogenesis of chicken adipocytes [65], suggesting a conserved role in fat deposition. miR-127 is highly expressed in mesenchymal stem cells and may affect cell differentiation into myotubes and adipocytes, including in mouse [66,67] and pig, respectively [68]. As a widely conserved miRNA, it is likely to be involved in fat and protein deposition in cattle. It has also been associated with disease, such as bovine lameness and mastitis [28], which could influence milk yield. It is interesting that both miRNAs are involved in adipogenesis and that the relative proportion of the two miRNAs was associated with milk fat and protein yield in the present study. This could be due to the negative association of adipogenesis with miR-127 [68] and positive association with miR-30c-5p [63,65]. This implies greater combined than individual effects of the two miRNAs on cow performance.

The ratio of miR-154b: miR-127 was positively associated with second lactation protein yield. There is scarce literature on these miRNAs in cattle, which does not always suggest involvement in milk protein. Wang et al. [69] showed that miR-154b expression in mammary tissues of cows was associated with milk fat and protein content. This miRNA requires further research in cattle to elucidate its function and potential role in milk protein yield.

Our results demonstrated that the change in miRNA levels over time may be of greater significance to cattle performance traits than single miRNA measurements. The fold change in miR-126-3p from young calf to first lactation cow was significantly associated with milk yield across the first two lactations of the cow. Previous research has linked miR-126-3p with lipid production and deposition, which may be related to milk production. Specifically, miR-126-3p was associated with lipid synthesis in mouse mammary epithelia [70], and was differentially expressed in the mammary glands of lactating goats undergoing food deprivation [71] and in the skeletal muscle of beef cattle of differing fatty acid composition [72]. Additionally, this miRNA has been associated with tissue angiogenesis [73]. Mammary tissue becomes more vascularized during pregnancy and lactation, followed by involution [74], and cows with higher mammary blood flow had higher milk yield [75]. Hence, the effects of miR-126-3p on vascular function may contribute to mammary tissue development during calf growth and/or when milk production is initiated during cow pregnancy and early lactation. However, the role of miR-126-3p depends on context; for example, this miRNA boosts differentiation of embryonic stem cells into endothelial cells but reduces proliferation in mature endothelial cells [73]. Therefore, further experiments investigating the role of this miRNA in different mammary-derived cell types and at different stages of animal life would provide new insights.

We performed cluster analysis of animals based on their miRNA levels, revealing differences between individuals in the trajectory of miRNA expression over time. For miR-126-3p, these clusters were significantly associated with second lactation SCC, a (sub)clinical mastitis indicator. In previous studies, the opposite strand (miR-126-5p) was reportedly downregulated in the mammary gland during mastitis episodes [76]. miR-126-3p is also elevated during pregnancy and lactation in mouse mammary epithelia and cow plasma [70,77,78], and is differentially expressed in the bovine corpus luteum during the oestrus cycle [79]. These previous studies identified expression changes in miR-126-3p during pregnancy, lactation, and infection, but here we identified associations of longitudinal measurements of miR-126-3p with milk production and udder inflammation. Additionally, a statistical trend was identified between calf levels of miR-126-3p and first lactation SCC. This could occur if this miRNA is involved in the development of mammary tissue and immunity, with possible effects on milk yield and infection risk, respectively, later in life. Further studies should investigate the role of miR-126-3p in postnatal and pubertal development in cattle.

In all the above analyses, we implemented a Bonferroni multiple testing correction based on 16 independent tests. This number was derived from four miRNA groups emanating from the miRNA relationships analyses and four cow performance trait groups corresponding to milk production, health, fertility and survival. Admittedly, this correction may have been stringent, as all miRNAs are eventually intercorrelated and all cow performance traits investigated are in some way related to each other. Therefore, we believe that results that remained significant after correction are true reflections of the associations identified.

In summary, building on previous data on early life performance biomarkers in cattle, we used longitudinal measurements in cows to report associations between the expression of several miRNAs, ratios, fold changes, and clusters with milk yield and composition, udder health, and fertility in the first two lactations of dairy cows. These promising associations suggest that these miRNAs warrant validation in further studies as a potential source of predictive biomarkers.

Further research should aim at better defining the functions of these miRNAs in cattle and quantifying their value as predictors of lifetime performance. For example, quantifying these miRNAs in a large sample size and performing cross-validation analyses with bootstrapping would investigate their predictive capacity. Further study is required to. elucidate the physiological bases proposed for the associations found in this study. Examples based on the above results could include studying the associations between miR-126-3p levels and mammary vascularisation or the effect of miR-126-3p, miR-30c-5p, or miR-127 on mammary epithelial cell function, as they were related to milk production traits. Studying correlations between miR-142-5p levels and postpartum metabolic changes would be warranted due to its association with calving interval.

## Supporting information

S1 File Figures S1-S13 demonstrating associations between miRNA measurements and cow performance traits that remained significant (P < 0.05) after adjusting for multiple testing. (PDF)
