# Supplementary material for: Dairy cow performance is associated with longitudinal microRNA profiles
Source: PLoS One. 2025 Aug 1;20(8):e0328765. doi: 10.1371/journal.pone.0328765 (PMC12316255; doi:10.1371/journal.pone.0328765)
Supplement: S1 File — (PDF) [file pone.0328765.s001.pdf]

S1_File

Figures S1-S13 demonstrating associations between miRNA measurements and cow performance traits that remained significant (P<0.05) after adjusting for multiple testing.

Associations with cow performance traits in the first lactation

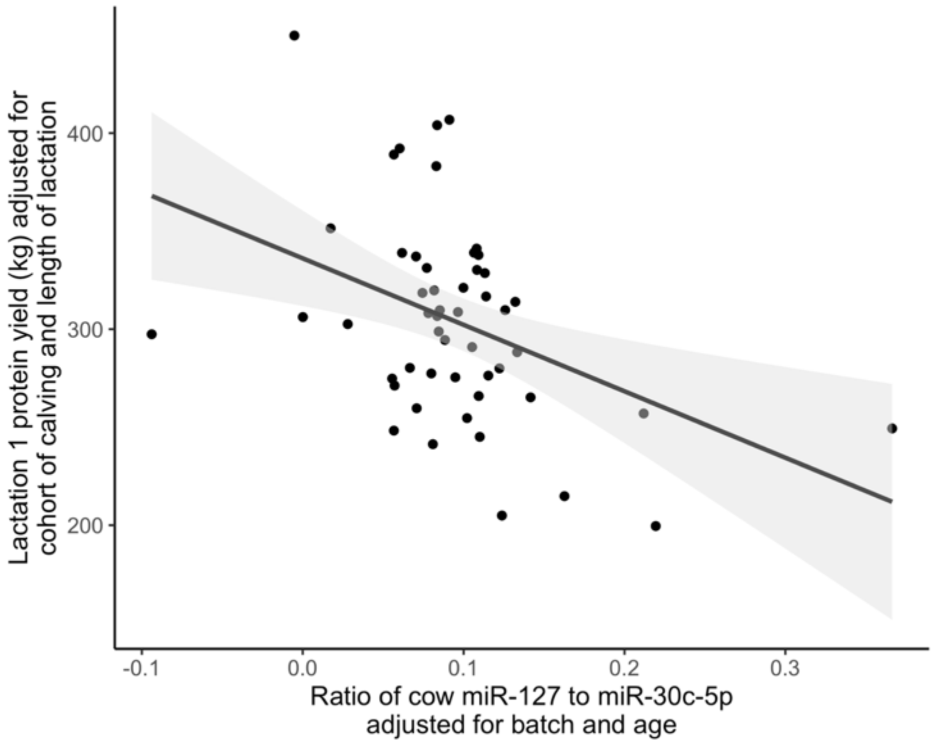

Fig S1: Scatterplot with linear regression and standard error for adjusted cow ratio level of miR-127 : miR-30c-5p against first lactation protein yield after adjusting for other factors in the model.

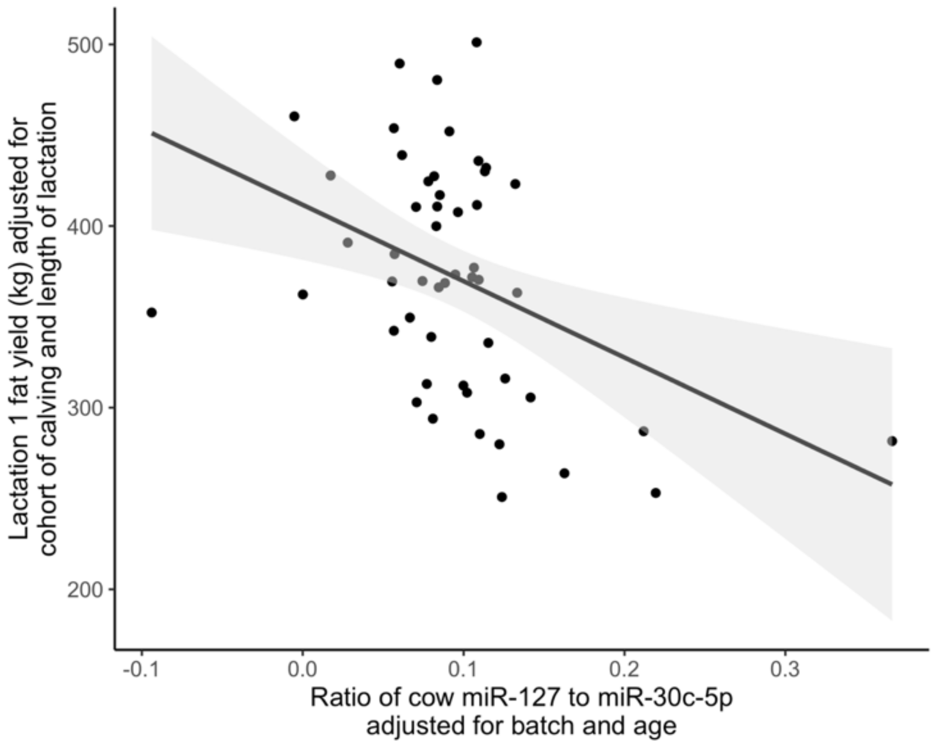

Fig S2: Scatterplot with linear regression and standard error for adjusted cow ratio level of miR-127 : miR-30c-5p against first lactation fat yield after adjusting for other factors in the model.

Associations with traits in the second lactation

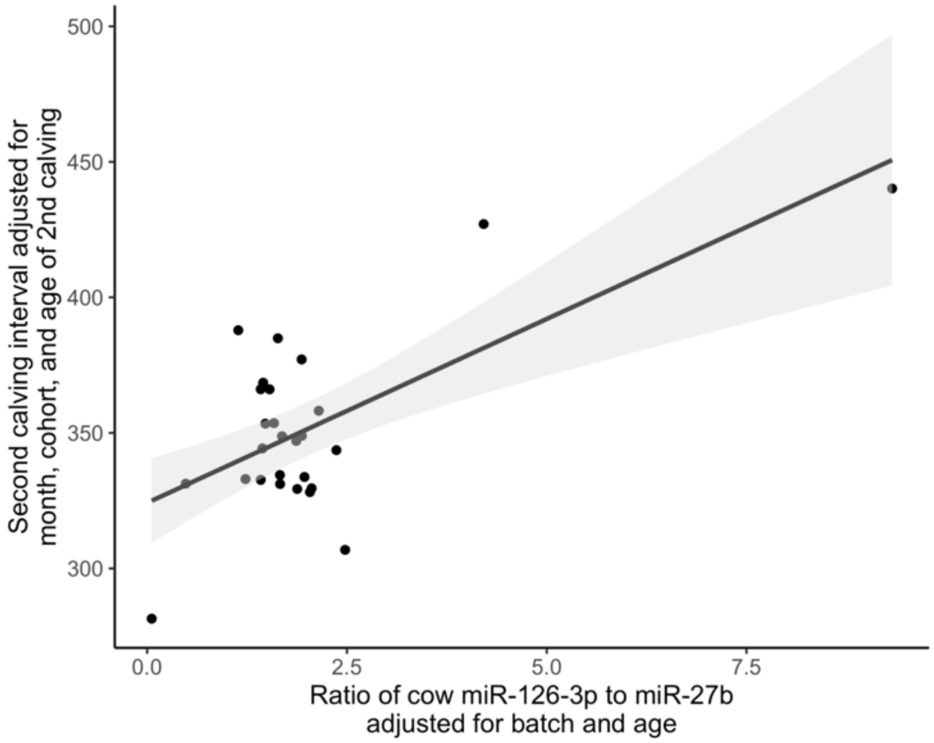

Fig S3: Scatterplot with linear regression and standard error for adjusted cow ratio level of miR-126 : miR-27b against second lactation calving interval after adjusting for other factors in the model.

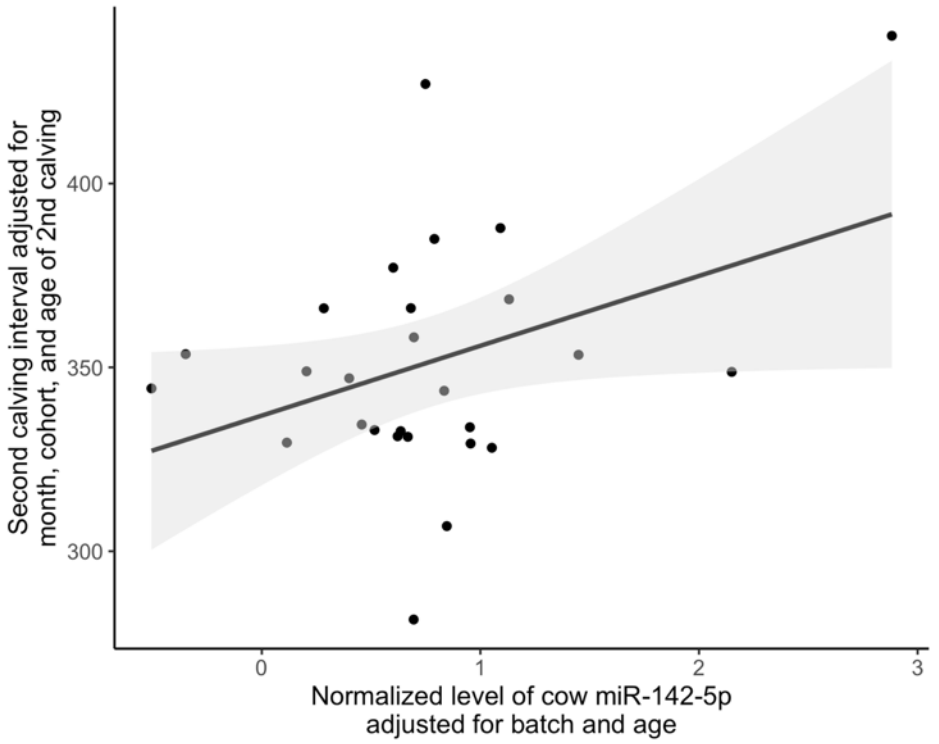

Fig S4: Scatterplot with linear regression and standard error for adjusted cow level of miR-142-5p against second lactation calving interval after adjusting for other factors in the model.

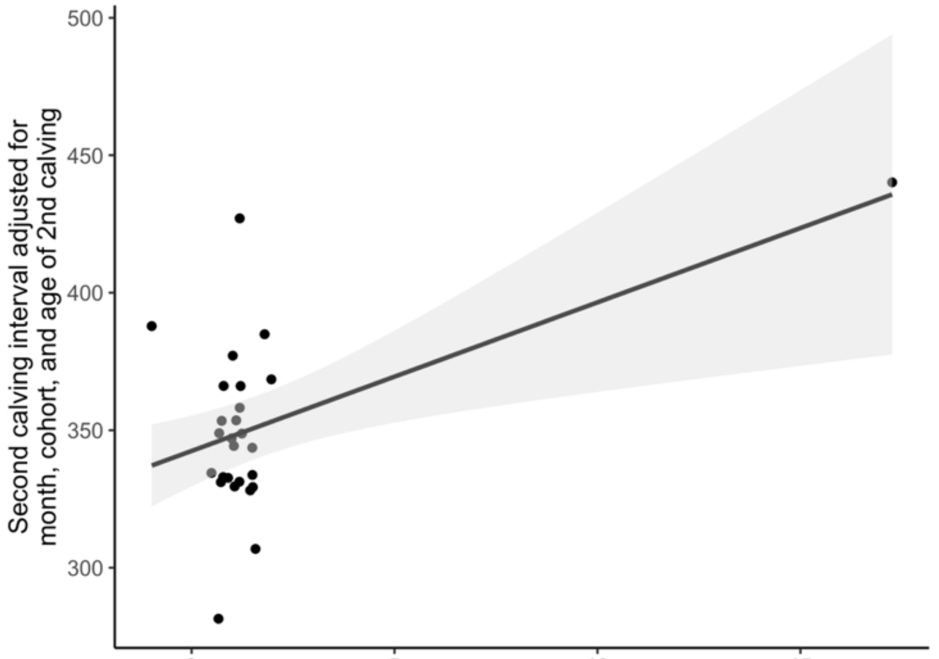

Fig S5: Scatterplot with linear regression and standard error for adjusted cow ratio level of miR-142-5p : miR-27b against second lactation calving interval after adjusting for other factors in the model.

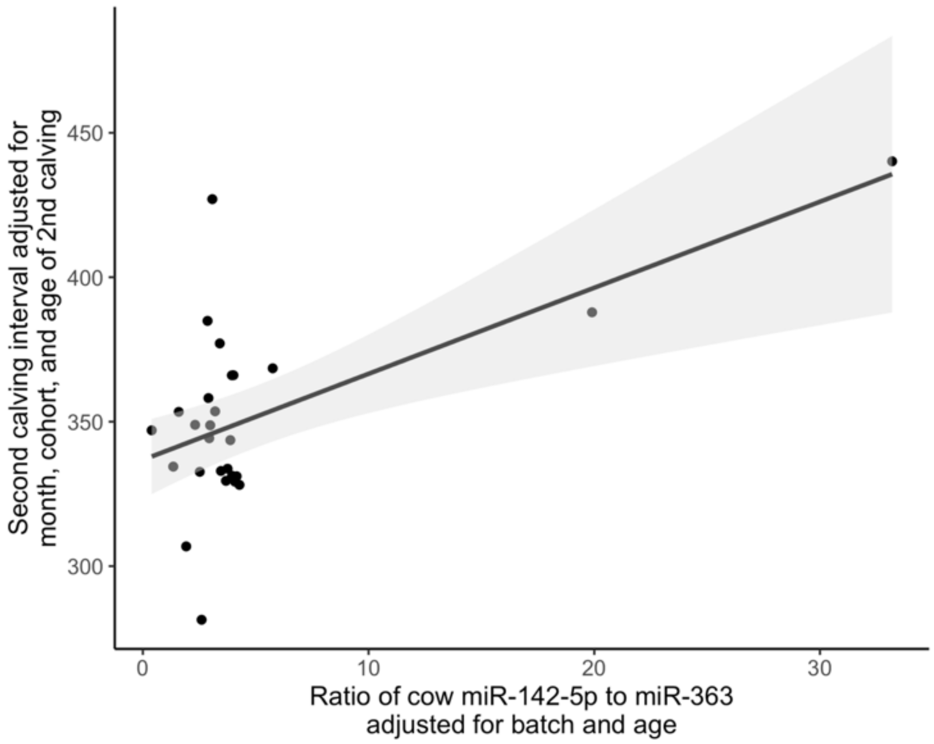

Fig S6: Scatterplot with linear regression and standard error for adjusted cow ratio level of miR-142-5p : miR-363 against second lactation calving interval after adjusting for other factors in the model.

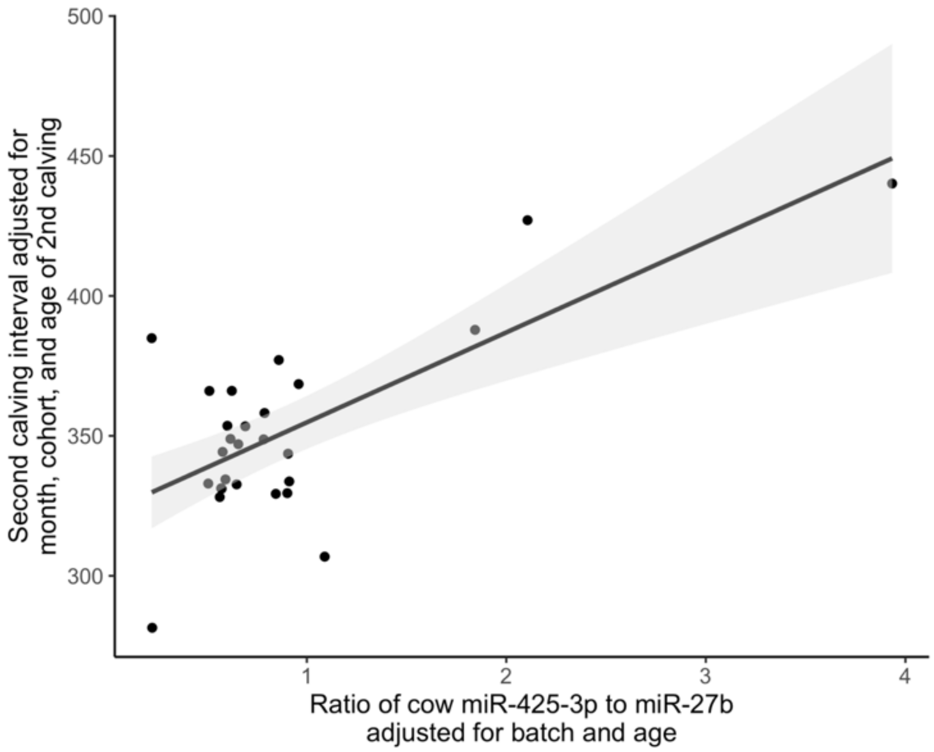

Fig S7: Scatterplot with linear regression and standard error for adjusted cow ratio level of miR-425-3p : miR-27b against second lactation calving interval after adjusting for other factors in the model.

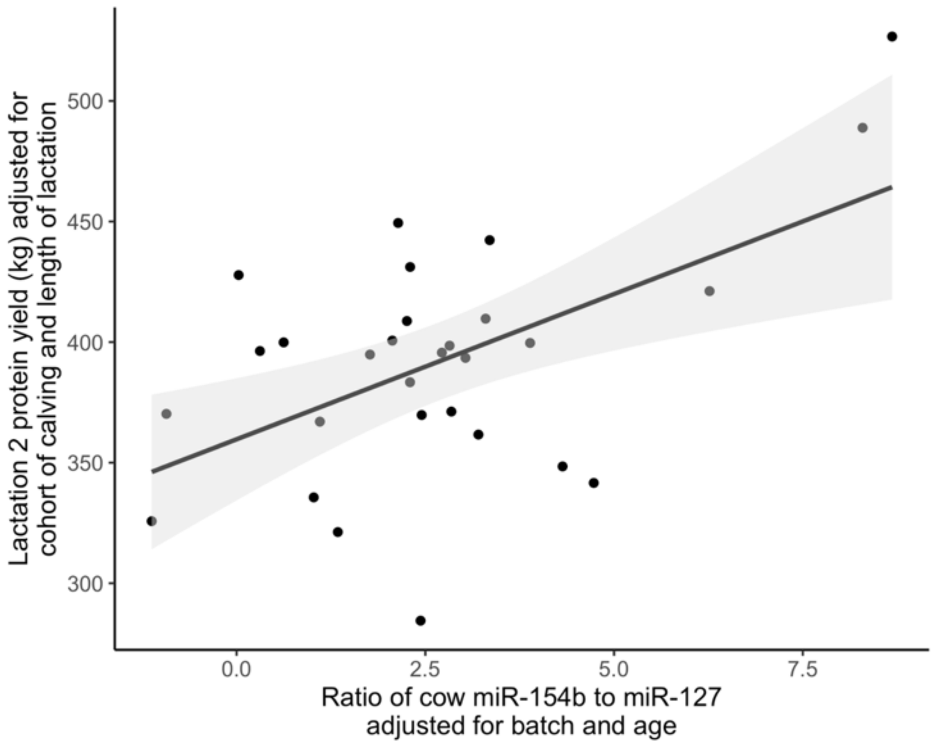

Fig S8: Scatterplot with linear regression and standard error for adjusted cow ratio level of miR-154b : miR-127 against second lactation protein yield after adjusting for other factors in the model.

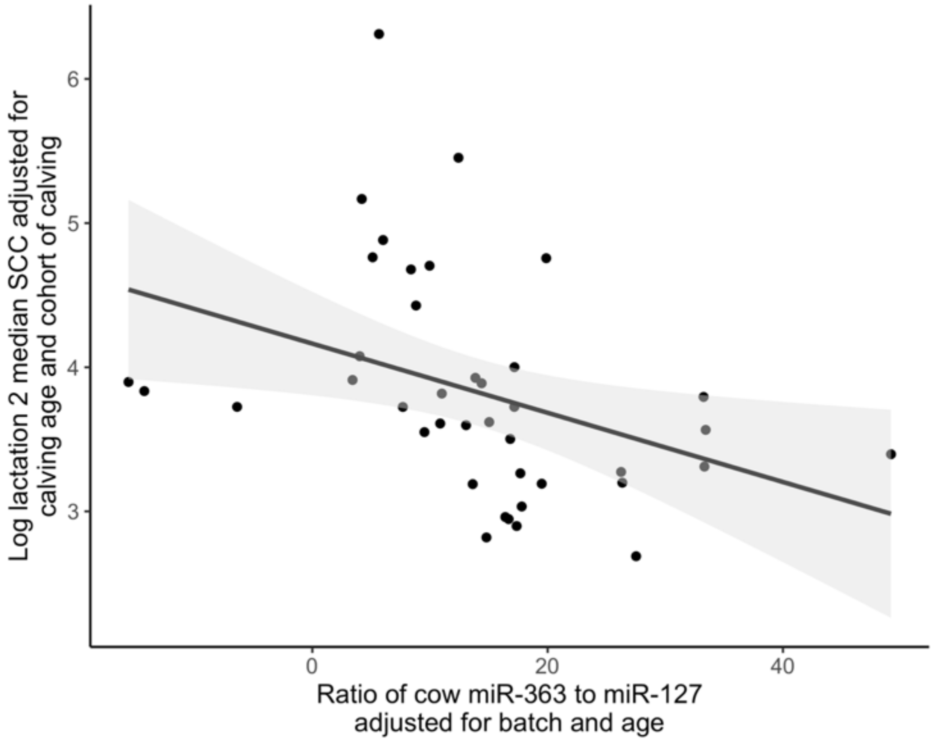

Fig S9: Scatterplot with linear regression and standard error for adjusted cow ratio level of miR-363 : miR-127 against second lactation log median SCC after adjusting for other factors in the model.

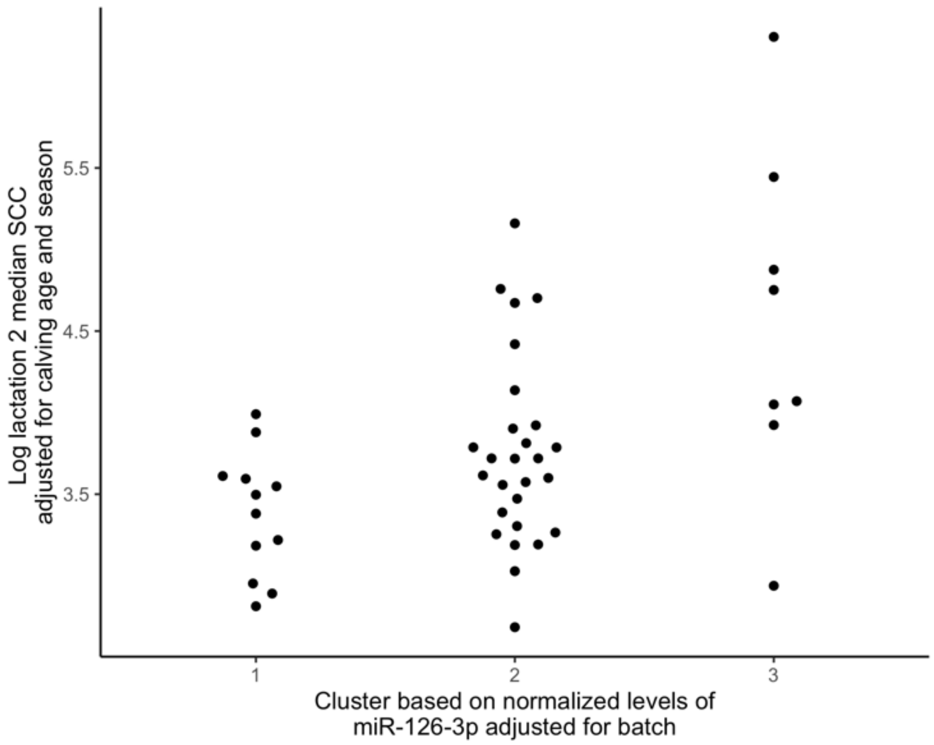

Fig S10: Swarm plot showing log median SCC after adjusting for other factors in the model, for each cluster based on miR-126-3p throughout life.

Associations with traits in the combined first and second lactations

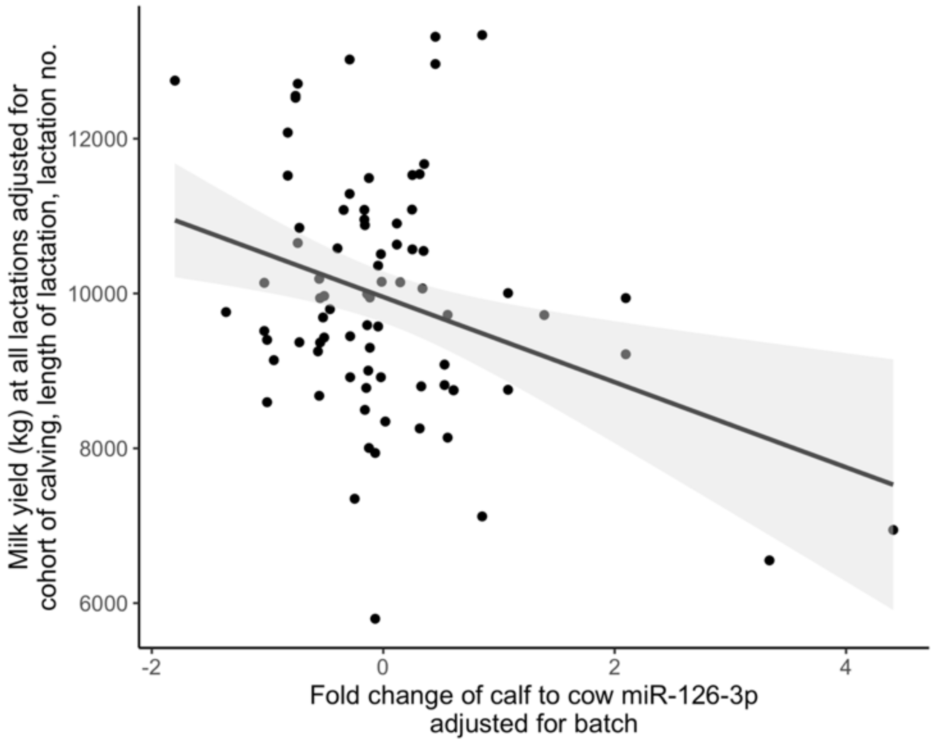

Fig S11: Scatterplot with linear regression and standard error for batch adjusted fold change of miR-126-3p from calf to first lactation cow against milk yield in the first two lactations after adjusting for other factors in the model.

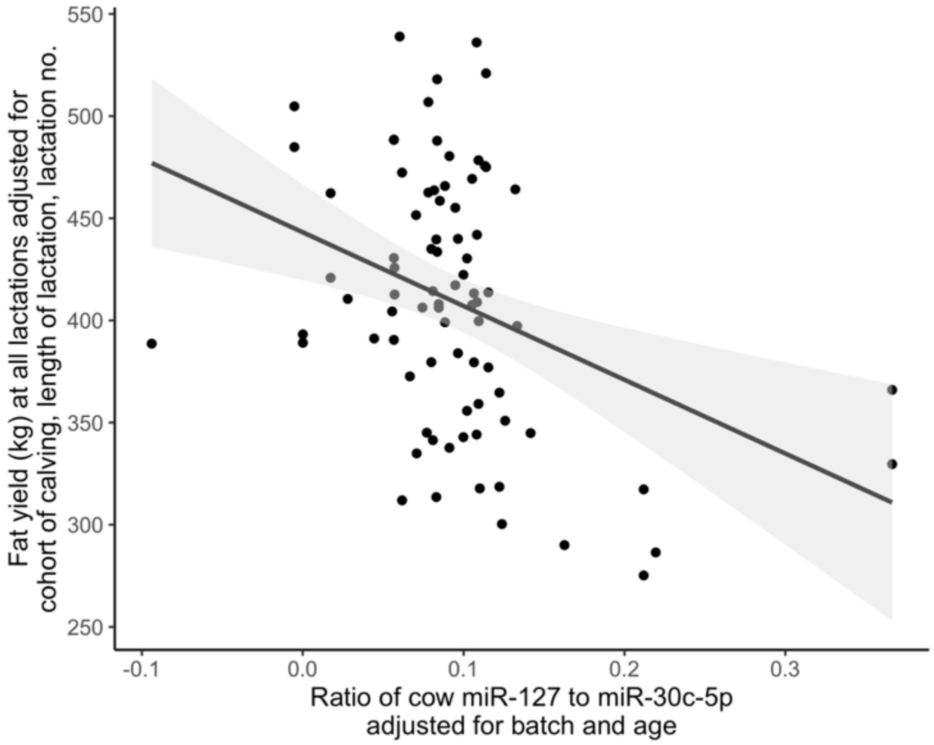

Fig S12: Scatterplot with linear regression and standard error for adjusted cow ratio level of miR-127 : miR-30c-5p against fat yield in the first two lactations after adjusting for other factors in the model.

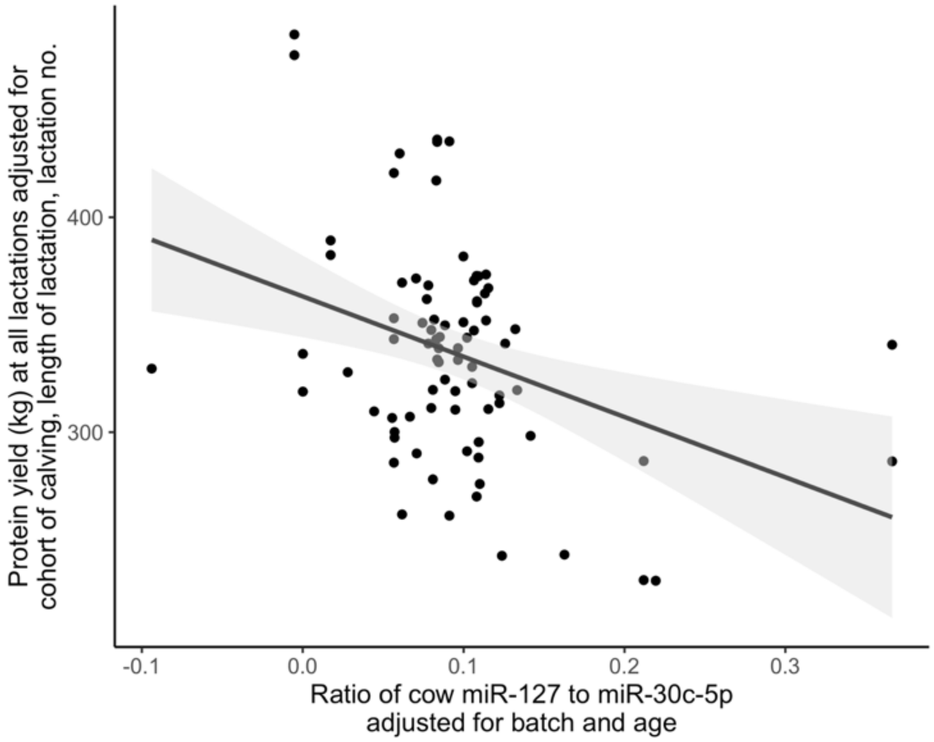

Fig S13: Scatterplot with linear regression and standard error for adjusted cow ratio level of miR-127 : miR-30c-5p against protein yield in the first two lactations after adjusting for other factors in the model.
